# Joints with angle dependent damping can help to reduce impact forces in robots

**DOI:** 10.1038/s41598-025-13055-7

**Published:** 2025-08-05

**Authors:** Shehara Perera, Saeed Bornassi, Mazdak Ghajari, Thrishantha Nanayakkara

**Affiliations:** 1 Dyson School of Design Engineering, Imperial College London, London, SW7 2DB UK; 2 Department of Engineering, University of Cambridge, Cambridge, CB2 1PZ UK

**Keywords:** Robotic joints, Angle-dependent variable damping, Drop test, Collision force mitigation, Mechanical engineering, Biomechanics

## Abstract

This paper investigates how a new angle-dependent damper design can help a robot to reduce collision forces. We designed a fluid-viscous angle-dependent damper by smoothly changing the clearance between the stationary and moving parts. Analytical and numerical simulation-based predictions were experimentally tested. Analytical modelling shows that angle-dependent damping has a $48\%$ reduction in peak forces when compared to constant damping. Numerical simulations show that variable-gap dampers can change damping by $134 \times$ during a $10 \times$ gap change. The experimental findings confirm the analytical predictions by reducing collision force by up to $10\%$. These findings suggest that the angle-dependent variable damping solution could be used for robots that experience collisions such as industrial robotic manipulators, legged robots, perching robots, or robots that catch moving objects.

## Introduction

Stable management of punctuated interaction forces such as in physical collisions remains a major challenge for robots. This challenge is shared across applications such as legged walking^1–3^, perching^4^, and catching moving objects^5^ where punctuated state transitions pose challenges for stability of the robot. Sato et al.^6^ used a soft-landing trajectory combined with optimal approach velocity to reduce impact force at the contact establishment. Their simulation results demonstrated that the robot can suppress the impact force while maintaining position control at the bottom. Calderon et al.^7^ conducted research with the goal of developing a control strategy for a simple robot to produce soft impact during the landing phase of a jump process. They proposed a method for reducing impact intensity based on the manipulation of compliance level and centre of mass. While high bandwidth control is an option to manage high frequency oscillations in states, a robot hardware design level solution for peak collision force minimisation is a better option to minimise the burden on feedback controllers.

Several hardware techniques, both passive and active, have been proposed to address these issues. A soft elastomeric pad is used at the foot sole in most cases and in the simplest way. This material acts as a shock absorber and can passively absorb the impact energy generated by a collision with the ground. Choi et al.^8^ introduced a new foot sole design aimed at improving the humanoid foot-leg capability in terms of reducing impact forces during landing and walking. A viscous air damping mechanism, consisting of three air-vessels implemented at the toe and heel sides of the foot, was considered in the design of the sole to provide energy absorption. Their findings show that the proposed foot sole design is effective at reducing ground impact forces while maintaining the robot’s stability in bipedal locomotion. Rond et al.^9^ created a foot design methodology that protects a legged robot from the negative effects of repeated ground impacts. They demonstrated that spring-damper coupling reduces rigid body collisions and foot vibrations. In fact, the spring at the bottom of the foot can gradually bring the leg to a stop during landing and allow it to touch down on the ground.

Another method for reducing impact intensity is to use compliant legs with passive elements at the joints. Choi et al.^10^ numerically investigated the effect of compliance on the leg-foot of a humanoid robot during landing impact, with the goal of reducing impact force through appropriate compliance tuning. They had found that a simple passive mechanism with a non-parallel foot posture during a landing from a certain height can effectively reduce impact forces. Moreover, Liow et al.^11^ showed that fibre jammed structures improves stability of robotic legs by varying stiffness and damping.

Despite passive techniques, active control can suppress impact force during the landing phase of legged walking^6,7,12–19^. Recently, Buchner et al.^20^ proposed a robotic leg built with electrohydraulic artificial muscles for soft landing with tunable stiffness. The majority of the research in this context is concerned with controlling the landing phase by controlling the landing velocity or rate of descent. Reduced impact velocity reduces impact force and thus provides a soft landing.

It is also worth noting that variable damping is a common solution for shock absorption in aircraft landing gears^21–25^. A variable orifice size damper ensures smooth landing by maintaining a constant profile for strut load throughout the touchdown process. For a hopping robot using active control, Sung and Youm^16^ realised that variable stiffness and damping are needed to achieve a soft landing. Variable stiffness and damping show a moderate increase in thrust force, whereas constant values show an impulsive force peak. Kamei et al.^17^ mathematically analysed the feasibility of the soft landing for hopping robot. They studied the existence of an exact solution for the equation of hopping robot motion. Lynch et al.^18^ examined the force and impedance control algorithms to minimise the foot penetration into a yielding terrain for a given velocity. Zhang et al.^19^ investigated the collision force reduction by controlling the knee joint angular velocity after the robot touched the ground.

Given that biological counterparts experience similar interactions, and yet manage to elegantly maintain stability, we turned to look closer at the knee joint as a bio-inspiration. An in-vivo study of the human knee joint^26^ revealed that increasing muscle contraction with the angle of flexion increases the stiffness and damping properties of the knee joint. This implies that the internal impedance of the knee joint is angle-dependent. Hamid et al.^27^ recently investigated the effect of an angle-dependent damping in a robotic joint on the peak collision force and its variability. Their results show that the hyperbolic angle-dependent damping pattern can reduce the magnitude and variability of the peak collision force.

In this paper, we present a novel passive physical method to implement angle-dependent variable damping to stabilise a robot experiencing collisions. This design leverages internal viscous damping within a joint in a compliant linkage to mitigate the occurrence of punctuated variations in states. By minimising the peak ground reaction force and its associated variability, this design aims to reduce the burden on closed loop controllers to stabilise robots under disturbances from collision. The key contributions of this paper are as follows:

1. Proposing a variable damping approach to attenuate the collision force: Passive implementation of variable damping in the joint for collision attenuation.
2. A novel variable rotary damper: Designing a viscous damper with adjustable damping to lessen the impact severity during impact.
3. Replicating the dynamic impact with ground with a drop test: Assessing the variable damper performance in mitigating the collision force in a vertical free fall drop test.

## Methods

In the context of a robotic end effector collision, complete cycle would involve contact establishment (impact), compression, restitution and recovery from collision. However, to investigate the effect of angle-dependent damping on collision forces, we focus on the contact establishment, compression and restitution. Therefore, each collision can be assumed to be analogous to a drop of the robot end effector from a certain height. Studies by Hwangbo et al.^28^ and Liow et al.^11^ employed drop tests to replicate dynamic legged locomotion, with the former utilizing simulations and the latter conducting experimental validation to demonstrate real-world applicability. Therefore, drop tests serve as an effective method for evaluating the dynamic behavior of an impact.

### Analytical modelling of angle-dependent damping

The utilisation of a multi-segmented model enables the anticipation and analysis of the dynamic aspects of a robot collision scenario. Therefore, in this study, we use a lumped mass two-segment model representing a legged robot as an end effector in collision to investigate the impact of angle-dependent damping on impact dynamics. Among the various collision events that may occur in robotic applications, we tested our proposed design on a robot leg-shaped structure to simulate the impact of a leg colliding with the ground.

#### Analytical model

**Fig. 1:**
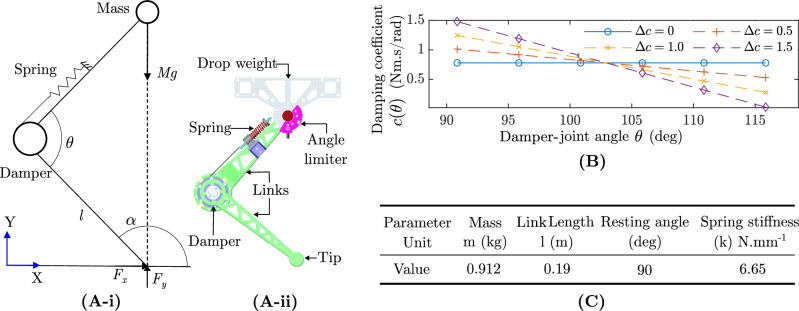
Analytical model: (**A**-i) Kinematic diagram of the segmented model. Entire mass is concentrated on top of the linkage. $F_y$ and $F_x$ are vertical and horizontal forces. The passive damper-joint is pinned and consists of a damper and a spring. (**A**-ii) 3D model of the system including the damper, spring, links and drop weight. (**B**) Angle dependent damping coefficient variation ($c(\theta )$) vs. Damper-joint angle ($\theta$). Plotted for mean damping coefficient $c =$ 0.78 Nm s $\text {rad}^{-1}$ and $\Delta c$ = 0, 0.5, 1.0, 1.5 Nm s $\text {rad}^{-1}$. (**C**) Analytical model parameters.

Figure 1A shows the two segment model in the sagittal plane and the equivalent 3D model of the experimental setup which will be discussed in the Sec. 2.2. The entire upper body of the robot is considered to be a concentrated point mass *m* on top of the upper link. The mass-less links having equal lengths *l* are connected by a pin joint acting as the damper-joint. A torsional spring with spring stiffness *k* and a damper with damping coefficient *c* at damper-joint represent the passive joint properties. $\theta$ is the angle between the links. $\alpha$ is the angle between the lower link and vertical axis.

Using Newton–Euler approach to derive the equations of motion for mass *m* we arrive at the following equations:

1 $$\begin{aligned}&\tau _{2} = k\Delta \theta +c\dot{\theta } \end{aligned}$$

2 $$\begin{aligned}&\tau _{2} +\tau _{1} = F_y l\sin {(\alpha +\theta )} - F_x l\cos {(\alpha +\theta )} \end{aligned}$$

3 $$\begin{aligned}&F_y - mg = m\ddot{y} \end{aligned}$$

4 $$\begin{aligned}&F_x = m\ddot{x} \text {,} \end{aligned}$$

where $F_y$ and $F_x$ are vertical and horizontal forces acting at the end of lower link. The parameter $\tau _{1}$ is the joint torque acting on the upper link and $\tau _{2}$ is the reactant torque at the damper-joint which occurs during the compression/extension of the linkage during motion. *y* and *x* are the vertical and horizontal distances to the mass *m* from the coordinated axis. $\ddot{y}$ and $\ddot{x}$ are the accelerations of the mass *m* in the Cartesian plane. Considering the damper and spring at the damper-joint, torque ($\tau _{2}$) can be calculated as

5 $$\begin{aligned} \tau _{2} = k\Delta \theta +c\dot{\theta } \text {,} \end{aligned}$$

where $\Delta \theta = \theta _{ref} - \theta$ with $\theta _{ref}$ being the angle at which spring force is 0, and $\dot{\theta }$ is the angular velocity of the damper-joint. For the case of linkage drop from a height, $\tau _{1} = 0$ and $\dot{x},\ddot{x} = 0$ and $F_x = 0$. Therefore, the above equations can be simplified to as

6 $$\begin{aligned} \ddot{y} = \frac{k\Delta \theta +c\dot{\theta }}{ml\sin {\alpha }}-g \text {.} \end{aligned}$$

This equation describes the motion of the mass *m* during the drop. A geometric relation can be used to determine the relation between $\ddot{y}$ and $\dot{\theta }$ as

7 $$\begin{aligned} 2l^2(1-\cos {(\theta )}) = y^2 \text {.} \end{aligned}$$

The ground reaction force ($F_y$) depends on the phase of collision can be expressed as

8 $$\begin{aligned} F_y = {\left\{ \begin{array}{ll} \frac{\displaystyle y (k\Delta \theta +c\dot{\theta })}{\displaystyle l^2 \sin {\theta }} & \text {During collision phase}\ (y \le h_0) \\ 0 & \text {During flight phase}\ (y > h_0) \end{array}\right. } \text {,} \end{aligned}$$

where $h_0$ is the final resting height of the linkage during steady state.

#### Implementation

The dynamic model was implemented in MATLAB and solved numerically using the MATLAB ode89 solver to determine the dynamics of the linkage with damper during the drop. Furthermore, a variable time step was used. All simulations were performed on a computer with an Intel Xeon W-2295 processor (3.00 GHz) and 32 GB of RAM running on Windows 10.

Damping coefficient of the damper $c(\theta )$ was changed according to the below linear relationship

9 $$\begin{aligned} c(\theta ) = a\theta +b \text {,} \end{aligned}$$

where *a* represents the gradient, calculated as $a = \Delta c/(\theta _f - \theta _0)$, and *b* denotes the intercept. In this context, $\Delta c$ denotes the parameter utilised for adjusting the level of damping during the descent motion. The final and starting angles are denoted as ($\theta _f$) and ($\theta _0$).

### Design of the damper and experimental setup

**Fig. 2:**
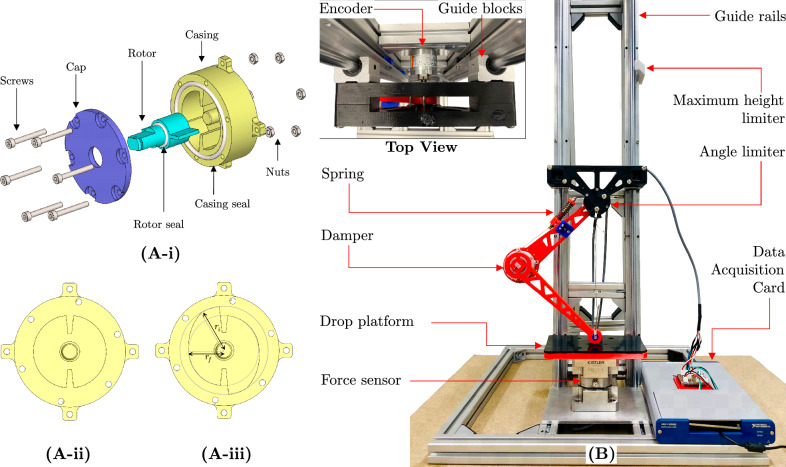
CAD model of the damper and experimental setup. (**A**-i) Exploded assembly of the damper including the cap, rotor and casing which are connected together using nuts and bolts while the seals are used to stop leakage of the damper oil. The inside volume of the damper is filled with damper oil which provides the required resistance for rotor movement. (**A**-ii) Top view of a constant damper casing. (**A**-iii) Top view of a variable damper casing which shows the clearance variation in the angle $\beta$. The initial casing radius is $r_i$ while the final radius is $r_f$. (**B**) Drop test rig with the linkage and sensor attachment for force measurement. Top view shows the encoder attachment in the free-rotating joint, which connects the upper link and drop weight, for angle measurement and guide blocks which slides on the guide rail.

The process of adapting a fluid-viscous damper to conform to a desired damping profile is a complex task, due to the turbulence of the fluid within the damper, which gives rise to non-linear behaviour. To address the intricacy and examine the impact of variable damping on mitigating ground reaction forces, a comparative methodology was employed in this study. The proposed methodology involved conducting an initial evaluation of a collection of dampers characterised by a consistent damping coefficient, followed by an examination of a separate set of dampers featuring a variable damping coefficient.

#### Damper design

A rotary vane damper design was selected to enable direct integration with a robotic joint. As depicted in Fig. 2A, the damper comprises of a casing, a rotor with vanes, and a cap.

When the rotary vanes undergo rotation within the fluid domain, the fluid is confined and can only exit through the narrow space between the tip of the rotor vane and the casing. As a result, this interaction generates a resistive force or torque, commonly referred to as drag, which opposes the rotational motion. The assumption is made that if the gap remains constant throughout the rotation of the vane, the resulting damping is also constant, referred to as a constant damper or constant gap damper. In order to introduce variability, adjustments were made to the clearance between the vane tip and casing, as depicted in Fig. 2A. In the case of variable dampers, specifically variable gap dampers, it should be noted that the radius of the casing on the inner side is not constant, but rather undergoes a change along a specific arc within a specified angle ($\beta$) as shown in the Fig. 2A from $r_i$ to $r_f$. The circular arc is tangent at $r_i$ to $r_f$.

#### Experimental setup

The damper is attached to the linkage in order to conduct drop tests aimed at quantifying the efficacy of the dampers. As depicted in Fig. 1, the linkage comprises of two distinct segments, namely the upper link and lower link, which are interconnected by means of a revolute joint referred to as damper-joint. The joint is equipped with a spring that provides the necessary stiffness, while a damper is installed at the revolute joint to provide the required damping. The upper link is connected to a holder that resemble free rotating joint. The holder is attached to the guide blocks and allowed to slide on the test platform, facilitating smooth and low-friction sliding during the drop test. To measure the damper-joint angle an incremental rotary encoder (Kubler KIH40 Series) was placed in the free-rotating joint as shown in the top view of Fig. 2B. The design presented here offers a passive structure that effectively emulates a robotic end effector.

The structural components were fabricated through the utilisation of Fused Deposition Modelling (FDM) 3D printing technology, employing Polyethylene Terephthalate Glycol (PETG) as the filament material. For testing, multiple dampers were fabricated and filled with silicon oil with viscosity ($\mu$) 100000 cSt. The total weight of the setup was 912 g and a compression spring with stiffness 6.65 N $\text {mm}^{-1}$ was used to provide the required joint stiffness.

The drop test rig consists of a platform constructed from aluminium that is securely fastened together using bolts. The linkage assembly is connected to the test platform via two guide rails (SBR12-1000) and bearing slider blocks (SBR12UU), as depicted in Fig. 2. The guide rails serve to restrict the movement of the linkage within the vertical plane. A load cell (Kistler triaxial type: 9367C) has been positioned beneath the drop plate to serve as a force sensor. The load cell is connected to a data acquisition unit (Kistler LabAmp type: 5165A). The encoder is connected to National Instruments data acquisition card. Both data acquisition units were connected to a computer.

To verify the effectiveness of a variable damping profile, physical properties of dampers such as effect of rotor-casing clearance, fluid viscosity, rotor velocity should be studied. Therefore, the next section presents a computational fluid dynamics analysis to quantify the angle-dependent damping behaviour of rotary-vane dampers.

### Computational fluid dynamics analysis

This section provides an analysis of the performance characteristics exhibited by both constant and variable rotary vane dampers through the utilisation of a Computational Fluid Dynamics (CFD) analysis.

#### Simulation model

**Fig. 3:**
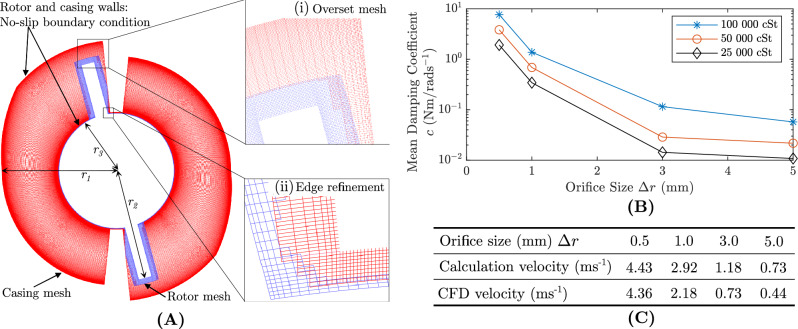
Computational fluid dynamics analysis: (**A**) Dynamic overset structured mesh grid of the casing and rotor is used in the computational fluid dynamics environment. (i): Overset mesh is used to transfer simulation results across two meshes, (ii) Edge refinements are added to resolve the flow near the walls of rotor and casing. (**B**) Computational Fluid Dynamics Simulation Results: Mean damping coefficient ($\bar{c}$) variation with the orifice size $\Delta r$ (rotor-casing clearance) plotted for three fluid viscosity values (25000 cSt, 50000 cSt and 100000 cSt). (**C**) Simulation results verification in comparison with analytical calculations using Eq. (13).

A numerical analysis was performed using ANSYS software to investigate the effect of casing geometry, rotor velocity, and fluid type on the damping coefficient. Since the rotor-casing geometry is uniform across 2D sections, the 3D geometry was simplified to a 2D geometry using ANSYS Design Modeller and used as the flow domain to perform the analysis. ANSYS fluent solver with transient formulation was used. To model turbulance, SST $k - \omega$ model was selected due to high viscosity of the fluid (low reynolds number flow) and its accuracy to model turbulance near boundaries. No-slip boundary conditions were imposed on casing and rotor walls. The flow domain is discretised into a grid as shown in Fig. 3 with a structured mesh using quadrilaterals to improve the convergence and to reduce numerical diffusion. Mesh refinements were added to resolve the fluid flow near the walls of the casing and rotor. Skewness was used as the metric to evaluate the mesh quality. The average mesh skewness values for casing and rotor were 0.07 and 0.08 and consisted of 155490 and 23810 mesh elements. A user-defined-function (UDF) was used to obtain the mesh motion of the rotor by specifying the rotor velocity. A pressure-based coupled solver was used as the pressure velocity coupling with relaxing higher order terms. In the solver, flow courant number was set to 100 while turbulent kinetic viscosity under-relaxation factor was set to 0.8 to improve the convergence.

The simulation was carried out by varying the following parameters:

- Casing geometry: constant dampers with casing-rotor clearances ($\Delta r$) of 0.5 mm to 5 mm and variable dampers with clearance variation of 3–0.5 mm. In variable dampers, clearance variation angle ($\beta$) was changed between $40^\circ$ to $60^\circ$.
- Fluid: Silicon oil with viscosities of 25000 cSt, 50000 cSt and 100000 cSt.
- Rotor Velocity: 20.944 rad $\text {s}^{-1}$, 10.472 rad $\text {s}^{-1}$, 5.236 rad $\text {s}^{-1}$, 2.608 rad $\text {s}^{-1}$ and 1.309 rad $\text {s}^{-1}$. The values were selected by comparing rotor velocities during experiments.

Calculation was initiated with an initial rotor position $\theta _i = 15^{\circ }$ and terminated at $\theta _i = 165^{\circ }$. Results are presented in Section “Computational fluid dynamics analysis”.

### Experimental evaluation of angle-dependent damping

To prove the effectiveness of variable damping empirically, drop tests were carried out. In the drop test, the linkage equipped with the damper and spring is released from a predetermined height.

#### Methodology

During experiments, following parameters were changed to study the effects of variable damping.

- Drop height: 80, 70, 60, 50 cm
- Damper: constant dampers (with clearance $\Delta r$ = 0.5, 1.0, 2.0, 3.0, 5.0, 6.0 mm) and variable dampers (with clearance $\Delta r$ = 5.0–0.5 mm, 3.0–0.5 mm, 2.0–0.5 mm and angle variation ($\beta$) from ${40}^\circ$, ${50}^\circ$ and ${60}^\circ$)
- Damper fluid: 100000 cSt

For each drop height and damper, the drop tests were repeated 10 times. In between two consecutive trials, a resting period of 5s was included between to eliminate transient dynamic effects such as post-impact oscillation and the unsteady fluid dynamics. The resting interval allows the system response to converge to a steady state condition by settling the transient effects. The ground reaction force and joint angle were measured using data acquisition cards and recorded using MATLAB. The signals were recorded with a high sampling rate (25 kHz) to capture the impact dynamics during the drop. The force and encoder signals were filtered first using a third-order one-dimensional median filter and then using a Savitzky-Golay filter with a polynomial order of 3 and a frame length of 101 to effectively mitigate noise in the data while preserving the overall shape and accuracy. Next, each trial was aligned with its first peak, and average and standard deviation of the 10 trials were calculated. The resulting curve was then subjected to post-processing analysis, which involved calculating the energy efficiency, peak force, and linkage deflection. The findings will be presented in the subsequent section.

The maximum ground reaction force ($F_{max}$) and the measurement of the maximum deflection ($s_{max}$) experienced by the linkage can be used as factors to evaluate the effectiveness of dampers. A decrease in the maximum ground reaction force serves to mitigate the potential for structural damage, while a reduction in lower link deflection results in a reduced level of physical constraint. The force sensor directly measures the load that is transmitted to the link. However, the calculation of linkage deflection can be derived by utilising the encoder data, given as

10 $$\begin{aligned} s_{max} = {h_0} - l\sqrt{2\left( {1 - \cos \left( \theta _{max} \right) } \right) } \text {,} \end{aligned}$$

where $\theta _{max}$ is the damper-joint angle during maximum compression, as shown in Fig. 2B, and $h_0$ is the initial height of the linkage before collision which can be obtained as ${h_0} = \sqrt{2} \,l$.

The evaluation of damper performance involves minimising ground reaction force while simultaneously optimising energy absorption efficiency and minimising damper-joint angle (or linkage contraction/linkage deflection) variation. The aforementioned methodology is widely used in the domain of shock strut design for aircraft landing gear^22^.

In this paper, we argue that the efficiency of collision response of a joint is high if the contact force profile during the collision remains flat without marked peaks. Therefore we define the efficiency of collision response as the the ratio between the area under the force-deflection curve ($\int _{0}^{s_{max}} F(s)ds$) and the area corresponding to the maximum reaction force ($F_{max}s_{max}$).

More specifically, the collision response of a joint is defined as

11 $$\begin{aligned} \eta = \left( \frac{\int _{0}^{y_{max}} F(s)ds}{F_{max}s_{max} } \right) \text {.} \end{aligned}$$

The variable $\eta$ provides insight into the energy absorption capacity of the linkage incorporating the damper and spring.

## Results

### Analytical modelling of angle-dependent damping

**Fig. 4:**
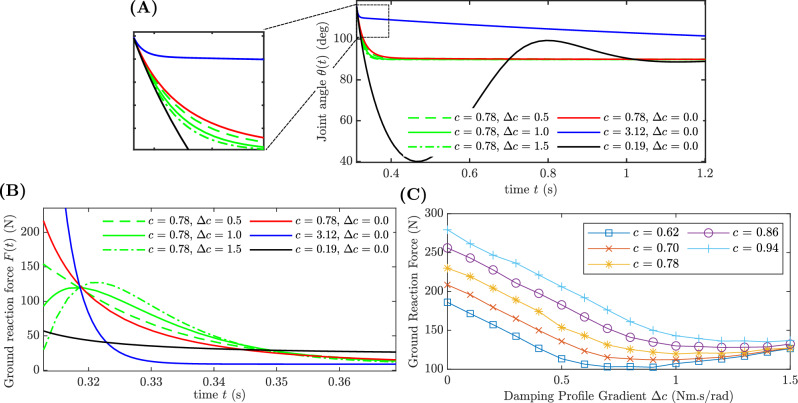
Analytical results of the linkage with damper. (A) Damper-joint angle vs. time for constant damping (with damping coefficients: critically-damped: 0.78 Nm s $\text {rad}^{-1}$, over-damped: 3.12 Nm s $\text {rad}^{-1}$ and under-damped: 0.19 Nm s $\text {rad}^{-1}$) and variable damping profiles (with profile gradients: $\Delta c =$ 0.5, 1.0, 1.5 Nm s $\text {rad}^{-1}$ and mean damping coefficient $c =$ 0.78 Nm s $\text {rad}^{-1}$). (B) Ground reaction force vs. time for constant damping (with damping coefficients: critically-damped: 0.78 Nm s $\text {rad}^{-1}$, over-damped: 3.12 Nm s $\text {rad}^{-1}$ and under-damped: 0.19 Nm s $\text {rad}^{-1}$) and variable damping profiles (with profile gradients: $\Delta c =$ 0.5, 1.0, 1.5 Nm s $\text {rad}^{-1}$ and mean damping coefficient $c =$ 0.78 Nm s $\text {rad}^{-1}$). (C) Maximum ground reaction force vs. damping profile gradient $\Delta c$ plot. Five mean damping coefficients *c* (0.62, 0.70, 0.78, 0.86 and 0.94 Nm s $\text {rad}^{-1}$) were used.

Figure 4A shows the variation of joint angle with time for six damping profiles (three constant damping profiles and three variable damping profiles). It can be seen that for constant under-damped damping case, the response oscillates. For constant over-damped case, the response requires a long time to reach the steady state value. The constant critically-damped case settles faster without oscillatory behaviour. Figure 4B shows the variation of the ground reaction force vs. time for six damping profiles. It can be seen that for constant damping profiles when the damping coefficient is in the over-damped region, the initial ground reaction force is significantly high. When the critical-damping is reached, the ground reaction force reduces (230.00 N) and is further reduced in the under-damped region (58.35 N). For variable damping profiles when the gradient ($\Delta c$) is increased from 0 to 1.5 Nm s $\text {rad}^{-1}$ the ground reaction force profile changes and results in a peak during the compression of the spring. Due to this change in the force profile, the maximum ground reaction reduces up to 120.13 N for $\Delta c =$ 1 which is a 48% reduction compared to the constant critically-damped case. When the gradient is increased further, the initial ground reaction force reduces. However, the peak increases beyond the optimal case which results in a higher ground reaction force.

Figure 4C illustrates the relationship between the peak ground reaction force and the gradient of the damping profile across six different scenarios. In all damping profiles, an increase in the gradient results in a parabolic pattern of the peak ground reaction force, characterised by a minimum value. When the mean damping coefficient deviates from the under-damped regime (where $c < 0.78$) towards the over-damped regime (where $c > 0.78$), there is a rightward shift in the minimum value. The observed parabolic pattern indicates that it is possible to optimise the variable damping profile in order to minimise the peak ground reaction force experienced during an impact.

### Computational fluid dynamics analysis

**Fig. 5:**
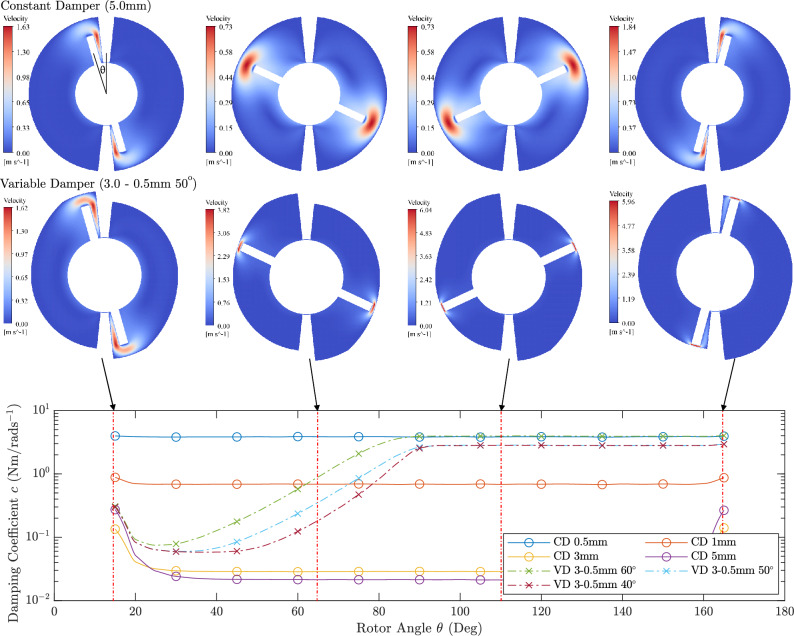
Computational Fluid Dynamics Analysis Results: Visualisation of fluid flow velocity (top) and damping coefficient variation (bottom) with time for both constant and variable dampers during rotor rotation. Flow visualisation is shown for four rotor positions ($\theta$): $15^\circ$, $65^\circ$, $110^\circ$ and $165^\circ$. Damping coefficient is shown for constant dampers with clearance $\Delta r = 0.5, 1, 3, 5$mm and variable dampers with clearance $\Delta r = 3 - 0.5$mm. The rotor-casing clearance was changed during the initial phase of the rotation, varying between $\beta = 40^\circ$, $50^\circ$, $60^\circ$ of the total rotation cycle..

The rotor torque was calculated in CFD-Post. Using rotor torque damping coefficient can be calculated as

12 $$\begin{aligned} C (\theta , \dot{\theta }) = \frac{\tau (\theta , \dot{\theta }).d}{\dot{\theta }} \text {,} \end{aligned}$$

where $C(\theta , \dot{\theta })$ [N m s/rad] is the damping coefficient which is a function of $\theta$ and $\dot{\theta }$, $\tau$ [N m/m] is the rotor torque, *d* [m] is the thickness of the rotor and $\dot{\theta }$ [rad/s] is the rotor angular velocity. Figure 3B shows the mean damping coefficient $\bar{c}$ of dampers with rotor clearances $\Delta r$ which varies from 3 mm to 0.5 mm. The mean damping coefficient was calculated using $\sum C (\theta , \dot{\theta })/n$. The three plots show the variation for silicon oil viscosities ($\mu$) 25000 cSt, 50000 cSt and 100000 cSt for 20.944 rad $\text {s}^{-1}$. It is evident from the figure that higher viscosity values have higher damping coefficients regardless of $\Delta r$. Also, when the $\Delta r$ decreases, the mean damping coefficient ($\bar{c}$) increases exponentially. For $\Delta r =$ 5 mm and $\mu =$ 100000 cSt, mean damping coefficient is 0.057 Nm s/rad and for $\Delta r =$ 0.5 mm it is 7.68 Nm s/rad which is an increase of $134\times$. When the viscosity is reduced by half to 50000 cSt, the mean damping coefficient is reduced by 0.5. A similar trend could be observed for 25000 cSt viscous silicon oil. Moreover, it was observed that changing the rotor velocity from 20.944 rad $\text {s}^{-1}$ to 1.309 rad $\text {s}^{-1}$ in the simulations had a negligible impact on the damping coefficient.

The flow velocity through the orifice can be analytically calculated as

13 $$\begin{aligned} v = \frac{\dot{\theta } ({r_2}^2 - {r_3}^2)}{r_1 - r_2} \text {,} \end{aligned}$$

where *v* is the flow velocity through the orifice, $r_1$ is the casing radius, $r_2$ is the rotor radius and $r_3$ is the rotor mid section radius. Figure 3C shows the comparison between simulation prediction and calculation using Eq. (13). In simulation flow velocity through the orifice is taken as the velocity of the mid point between rotor and casing. For small orifice sizes the two values are similar (2% difference). However, for larger orifice sizes the difference is 40%. This difference occurs due to non-uniform flow profile through the orifice.

Figure 5 shows the flow velocity visualisation across fluid domain and damping coefficient variation with time for constant dampers and variable dampers. From both constant and variable dampers, it can be observed that the initial flow at $15^\circ$ and final flow at $165^\circ$ is different from the flow domain at $65^\circ$ and $110^\circ$. This is also evident in the damping coefficient plot where initial and final damping coefficients are higher and is different from the other rotor positions. This difference can be associated to the unsteady fluid flow at the initial and final rotor positions as can be seen in the vortex flow patterns in these two positions. Due to this difference, the damping coefficient becomes non-linear and exhibits a non-linear behaviour around these two instances. This fluid flow difference affects the damping coefficient for approximately $15^\circ$ on both ends. For constant dampers, apart from the end non-linearities, the damping coefficient is constant for a rotor angle variation ($\Delta \theta = 135^{\circ }$) owing to higher steadiness of the fluid flow and uniformity.

For variable dampers, the non-linear behaviour is evident from the plot as shown in Fig. 5. When the rotor-casing clearance ($\Delta r$) was changed from 3mm–0.5mm within $60^\circ$, the damping coefficient increases. When the variable damper was changed from $60^{\circ }$ to $40^{\circ }$, damping coefficient changes within a smaller angle window and exhibits a sharp rise. However, it should be noted that in all cases, damping coefficient settles smoothly after orifice size reaches the constant value.

### Experimental evaluation of angle-dependent damping

#### Tip contact and passive damper joint dynamics

**Fig. 6:**
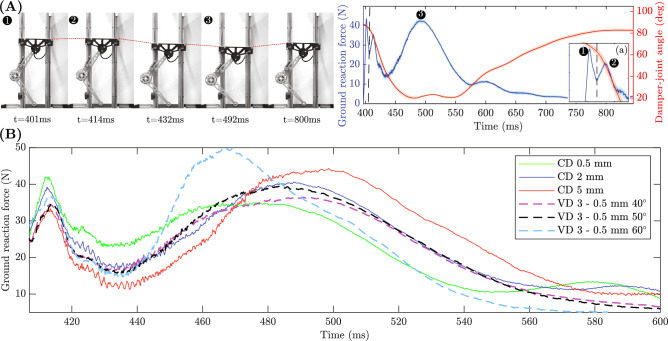
Experimental results: (**A**) Mean and standard deviation of the ground reaction force and joint angle measured during a drop test (shown for drop height = 80 cm and 3 mm constant damper). The initial peak (1) occurs due to the inertial effect of the tip contact dynamics while the remainders (2 and 3) corresponds to the spring and damper dynamics of the passive joint. The two regions are separated by a vertical dotted line as shown in zoomed section (a). Snapshots corresponding to different time points are shown: $t=$401ms corresponds to the (1) peak. $t=$414ms corresponds to the (2) peak. $t=$492ms corresponds to the (3) peak. (**B**) Ground reaction force variation over time in the region of joint dynamics for constant dampers (CD) and variable dampers (VD) for the drop height 80 cm. The plot shows the time interval in which the damper and spring are actively involved.

Figure 6 shows a typical force and angle profile for a drop test from a height 80 cm. It shows the mean and standard deviation of all trials performed for a 3 mm constant damper. The force plot reveals a sharp peak at the collision point, the moment at which the tip touches the force sensor. At the instant of collision, the damper angle has not been changed and it is fixed at the initial resting angle which is $90^\circ$. This sharp peak (shown as 1 in Fig. 6) is associated with the tip contact dynamics and is similar for all the drop tests with different dampers. This region is separated by a dotted line. Thereafter, spring and damper combination is engaged while the linkage deflects and can be associated to the dynamics of the passive joint. When spring and damper is engaged, it results in the second peak (shown as 2 in Fig. 6). The sudden drop in the reaction force after the second peak can be associated with the lift-off phase, the moment at which the linkage starts lifting-off from the ground surface or push back due to stiffness of the whole linkage and ground surface. The remaining part of the force curve is associated with the vibration of the linkage upon impact due to compliant elements in the system which produces a fluctuating force pattern. This is in agreement with the angle curve, showing oscillating pattern while the linkage vibrates. As seen, the linkage vibration is suppressed after a period of time and at the end approaches to a steady state condition.

**Table 1:** Performance of constant dampers.

Drop Height	Clearance ($\Delta r$)	0.5 mm	1 mm	2 mm	3 mm	4 mm	5 mm
80 cm	Max Force (N)	42.05 (1.05)	43.05 (0.53)	*** 40.59 (0.73)***	42.44 (0.99)	44.66 (0.70)	44.32 (1.71)
	Max Deflection (cm)	16.7 (0.3)	18.8 (0.9)	18.5 (0.8)	19.8 (0.4)	20.6 (0.2)	20.2 (0.2)
	Efficiency (%)	69.58	64.05	63.34	55.24	58.12	59.44
70 cm	Max Force (N)	39.67 (0.85)	40.34 (0.89)	35.41 (0.65)	*** 35.19 (0.79)***	38.81 (0.82)	38.70 (0.92)
	Max Deflection (cm)	15.3 (0.7)	16.6 (0.6)	17.4 (0.7)	19.8 (0.3)	20.0 (0.4)	20.0 (0.4)
	Efficiency (%)	68.18	64.47	68.43	60.49	49.38	56.39
60 cm	Max Force (N)	35.29 (0.67)	36.23 (0.85)	32.38 (0.70)	30.00 (0.72)	*** 29.51 (0.73)***	32.33 (1.82)
	Max Deflection (m)	12.8 (0.7)	13.7 (0.7)	15.1 (0.7)	18.6 (0.7)	19.5 (0.3)	19.3 (0.3)
	Efficiency (%)	70.17	67.09	68.23	66.53	63.86	55.3
50 cm	Max Force (N)	30.54 (1.09)	30.98 (1.02)	28.00 (0.69)	26.42 (0.61)	25.09 (0.78)	*** 24.65 (0.57)***
	Max Deflection (cm)	9.8 (0.7)	10.3 (0.8)	12.2 (0.9)	15.6 (0.6)	18.1 (1.1)	19 (0.5)
	Efficiency (%)	72.38	70.56	69.19	67.1	67.69	66.77

The standard deviation of trials is indicated within brackets for both the maximum force and maximum deflection. The dampers that demonstrate the lowest peak ground reaction force are emphasised using bold italics font.

#### Variation of ground reaction force between dampers

Tables 1 and 2 display the mean and standard deviation of the maximum ground reaction force, linkage deflection, and damper efficiency during the drop test conducted at heights of 80 cm, 70 cm, 60 cm, and 50 cm. The variable dampers can be divided into three sub-groups, each associated with a distinct set of patterns in gap variation by changing $\beta$. As previously mentioned, the current study places particular emphasis on the ground reaction force as an optimisation parameter. Nevertheless, the linkage deflection and damper efficiency were also documented in order to analyse their respective characteristics.

The constant damper with 2 mm clearance produces the lowest amount of peak ground reaction force for 80 cm. The variable damper 3–0.5 mm ${40}^\circ$ produces the lowest the ground reaction force for 80 cm drop height. This is equal to 10.20% reduction in the peak ground reaction force compared to damper with constant gap 2 mm. In contrast, for heights of 70 cm, 60 cm, and 50 cm, the lowest peak ground reaction force is almost the same for both constant and variable optimal dampers.

The remaining dampers within the constant damping group, which have lower and higher gaps, exhibit a higher peak ground reaction force. Indeed, when the gap is reduced, the damping increases and when the gap is increased, the damping reduces. As a result, increased damping leads to more forceful collisions, resulting in a higher peak collision force. This phenomenon is clearly visible in Fig. 6. The presence of a 0.5 mm gap in the damper results in a pronounced initial peak force during impact in the dynamics region, followed by a gradual decrease in force intensity over time. In contrast, decreased damping results in a more soft landing during the initial phase of impact, leading to a reduced peak collision force at the onset. Subsequently, a more prominent peak is observed, attributable to the presence of low damping and inadequate dissipation of kinetic energy. In practise, the optimal performance of dampers are typically observed within a range of approximately 0.5 mm to 5 mm gaps.

Another notable observation relates to the distribution of the ground reaction force in the vicinity of the third peak. Specifically, in the 3–0.5 mm ${40}^\circ$ damper results in a broader temporal dispersion of the ground reaction force. This is quantified by the ratio of the maximum force of the third peak to the difference in timestamps required to reach 0.8 times the value of the third peak force ($F_{max}/(t_{2-0.8 \times F_{max}}-t_{1-0.8 \times F_{max}})$). For the drop heights of 80 cm and 70 cm the highest metric value was 0.86 which was for the 3–0.5 mm ${40}^\circ$ compared to the constant damper which was 0.82. This suggests that the variable damper is capable of dampening the collision force throughout a wider spectrum of time compared to constant dampers.

**Table 2:** Performance of variable dampers.

Drop Height	Clearance ($\Delta r$)	5.0–0.5 mm			3.0–0.5 mm			2.0–0.5 mm		
	Angle Variation ($\beta$)	$40^\circ$	$50^\circ$	$60^\circ$	$40^\circ$	$50^\circ$	$60^\circ$	$40^\circ$	$50^\circ$	$60^\circ$
80 cm	Peak Force (N)	44.31 (4.14)	42.98 (1.86)	44.26 (2.03)	*** 36.45 (1.38)***	39.48 (1.73)	49.99 (7.36)	39.15 (1.29)	39.88 (5.30)	38.88 (3.85)
	Max Deflection (cm)	17.8 (0.5)	18.7 (0.3)	19.2 (0.8)	20.7 (0.3)	20.7 (0.5)	18.6 (0.8)	19.7 (0.6)	20.1 (0.5)	20.2 (0.5)
	Efficiency (%)	68.9	67.16	63.04	65.12	62.32	68.82	69.45	65.64	65.8
70 cm	Peak Force (N)	37.48 (3.13)	37.79 (3.42)	37.73 (1.26)	*** 34.31 (3.63)***	34.70 (3.50)	39.07 (5.40)	35.93 (0.80)	35.80 (4.79)	38.31 (3.67)
	Max Deflection (cm)	17.6 (0.7)	18.0 (0.8)	19.2 (0.3)	20.0 (1.1)	20.4 (0.2)	19.9 (0.7)	19.5 (0.8)	19.6 (0.9)	19.3 (0.8)
	Efficiency (%)	61.57	58.83	57.75	66.13	63.26	55.24	69.81	67.71	63.38
60 cm	Peak Force (N)	30.04 (1.11)	30.74 (1.00)	31 (0.69)	30.27 (1.07)	*** 29.83 (0.76)***	31.90 (2.71)	33.85 (1.22)	33.27 (0.66)	32.61 (0.59)
	Max Deflection (cm)	17.9 (0.011)	18.0 (0.3)	19.1 (0.3)	19.0 (0.3)	19.6 (0.3)	19.8 (0.3)	18.3 (1.1)	18.7 (0.2)	18.7 (0.3)
	Efficiency (%)	71.45	67.82	67.08	69.25	68.47	62.18	68.87	66.38	67.09
50 cm	Peak Force (N)	25.43 (1.02)	25.39 (0.65)	25.55 (0.61)	25.65 (0.59)	*** 24.86 (0.92)***	25.11 (0.62)	27.92 (1.08)	27.37 (0.50)	27.37 (0.53)
	Max Deflection (cm)	15.7 (1.3)	16.6 (0.8)	15.6 (1.2)	18.0 (0.6)	17.3 (1.6)	18.4 (0.8)	14.8 (1.7)	16.4 (1.1)	16.9 (0.6)
	Efficiency (%)	72.22	72.22	70.56	72.21	72.23	70.54	71.73	71.23	70.15

The standard deviation of trials is indicated within brackets for both the maximum force and maximum deflection. The dampers that demonstrate the lowest peak ground reaction force are emphasised using bold italics font.

#### Force transmission to the upper body

**Fig. 7:**
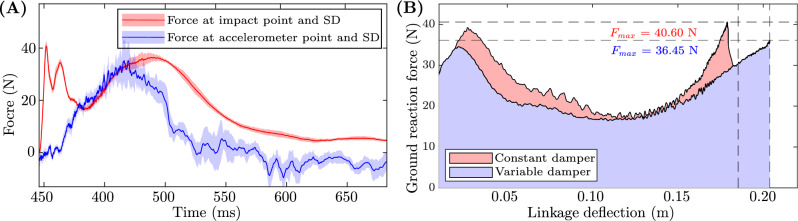
Force plots: (**A**) Force vs. time curve of 3–0.5 mm (${40}^\circ$) variable damper for 80 cm drop. Two curves show the force measured at the impact plate from the force sensor and the force estimated using an accelerometer attached to drop weight. (**B**) Ground reaction force vs. linkage deflection of two optimal constant and variable dampers for 80 cm drop.

The force variation for an 80 cm drop test of the setup equipped with a 3–0.5 mm ($40^\circ$) damper is illustrated in Fig. 7A. The first curve (in red) represents the force measurements obtained from the force sensor utilised in the drop test setup. The second curve (in blue) represents an estimation of the force exerted on the drop weight, which was calculated using the acceleration of the drop weight. The presence of the first and second peak in the force sensor data is not available in the accelerometer estimation. This implies that the incorporation of a passive joint with a damper functions as a filtering mechanism to mitigate the occurrence of abrupt initial peaks that arise during impact.

#### Impact absorbing efficiency

The force deflection curves for two test cases with 3–0.5 mm ($40^\circ$) variable damper and 2 mm constant damper are shown in Fig. 7. Both of these dampers are deemed optimum for the drop height of 80 cm. The curve shows how the ground reaction force varies when the linkage is compressed during the collision along with linkage deflection/damper compression. The observed curve indicates that the force profile associated with the optimal variable damper is comparatively lower than that of the constant damper, despite exhibiting greater linkage deflection. The area under the curve represents the measure of effectiveness of the damper. A higher degree of plateauing in the force curve corresponds to an increased level of efficiency in the damper. In general, variable dampers exhibit higher efficiency compared to optimal constant dampers. Specifically, the percentage increase in efficiency values for variable dampers are 2.80% for a height of 80 cm, 9.30% for a height of 70 cm, 7.20% for a height of 60 cm, and 8.90% for a height of 50 cm. The average efficiency increase observed across all heights is 7.1%.

## Discussion and conclusion

### Discussion

#### The effect of rotor-casing clearance on ground reaction force

Our objective is to leverage the benefits of constant dampers featuring both high and low damping characteristics to develop a damper with adjustable clearance. This design aims to mitigate the reaction force profile during the engagement period of the damper. This entails the development of a damper that can consistently generate a force profile that remains relatively constant over an extended period. The superiority of the variable damper 3–0.5 mm ($40^\circ$) over the constant dampers is evident in Fig. 7. As observed, the optimal variable damper initially generates a low force (at $t =$ 415 ms) owing to the significant clearance, and as the clearance reduces over time, the resultant force gradually converges towards a lower magnitude (at $t =$ 490 ms). This behaviour is evident from the computational fluid dynamics simulation results presented in the Sec. 3.2 which show that a higher gap ($\Delta r =$ 5 mm) results in very low damping ($c =$ 0.057 N m s/rad) and a lower gap results in very high damping ($c =$ 7.68 N m s/rad)

Another notable observation from Tables 1 and 2 is the negligible difference between optimal fixed and variable dampers in terms of force reduction at lower fall heights. However, as the fall height and the corresponding impact intensity increase, the advantage of variable damping in mitigating impact forces becomes more pronounced. This trend suggests that the efficiency of variable dampers surpasses that of fixed dampers under conditions of higher impact intensity.

#### The effect of drop height on damper selection

Experimental results in Table 1 show that variations in height result in alterations to the optimal damper selection for constant dampers. It is not possible for a single constant damper to effectively reduce impact forces across all heights. However, results in Table 2 show that the variable damper with a clearance reduction from 3 mm to 0.5 mm and angles of $40 ^\circ$ or $50^\circ$ degrees demonstrates approximate optimality across all drop heights. This implies that the variable damper is more robust and applicable compared to the constant damper.

#### Optimising a damper for optimal performance

To optimise shock absorber performance, the objective function should include peak force, linkage deflection, and damper efficiency. In the current study, as previously indicated, our focus is directed towards the reduction of the reaction force. Upon examining the data presented in Tables 1 and 2, it becomes evident that the dampers, whether constant or variable, deemed optimal, do not necessarily yield the most favourable outcomes in terms of linkage deflection and efficiency. The optimal constant damper exhibits a reduction in lower link deflection by $16\%$ when compared to the optimal variable damper. Nevertheless, the variable damper exhibits a slightly higher level of efficiency compared to the optimal constant damper. The damper with a constant gap of 0.5 mm is considered to be the most optimal choice due to its lower deflection and maximum efficiency, however, when it comes to peak collision force, it is not as effective as variable dampers. In contrast, the variable damper with a range of 5–0.5 mm ($40^\circ$) exhibits the least amount of linkage deflection. Additionally, the variable dampers with ranges of 5–0.5 mm ($40^\circ$), 3–0.5 mm ($60^\circ$), and 2–0.5 mm ($40^\circ$) are characterised by high efficiency.

The findings indicate that the utilisation of a variable damper at the joint has the potential to enhance the energy efficiency of the linkage during collisions while simultaneously mitigating the impact load to lower magnitudes. Compared to the constant dampers, the variable dampers appear to optimize impact mitigation by distributing the damping force over the full stroke of the damper, thereby delivering a minimized and relatively constant force with maximal efficiency. This functionality is, in fact, achieved by modulating the gap between the rotor and stator, thereby altering the damping characteristics throughout the damper’s stroke. As a result, the system operates in a semi-active manner, dynamically adjusting the damping response in proportion to the applied reaction force. Upon examination of the Tables 1 and 2, it becomes evident that the variable dampers exhibit greater energy efficiency when subjected to lower levels of impact force. The aforementioned observation confirms that variable dampers at the joints are best suited to reduce collision forces for applications such as legged robots, perching robots or robots that catch moving objects.

### Conclusion

We hypothesised that an angle-dependent damper in a joint of a robotic end effector would reduce peak force during a collision. The proposed approach aimed to gradually increase the dissipation of energy during a collision. Experimental results confirmed the analytical and numerical results that an optimal variable damper was capable of reducing the collision force by a maximum of 10.2% compared optimal constant damper. Across all drop heights optimal variable damper was able to increase the efficiency by 7.1%. Findings show that the utilisation of angle-dependent variable gap dampers can effectively mitigate the collision forces with higher efficiency.

In conclusion, our study presents a generalizable hardware-level solution to mitigate impulse forces, eliminating the need for sensors or active control strategies during impact dynamics. Rather than relying on an active controller to mitigate punctuated forces, our approach focuses on a hardware-based solution that inherently smooths out impact forces, ensuring robustness and practicality in a wide range of real-world robotic applications. This approach avoids the complexities associated with sensor-based feedback control, offering a straightforward yet effective means of stabilizing legged robots during collisions.

The performance of these variable dampers can be further improved by coupling them with springs for fast return back to initial conditions after collision. Future work will involve optimising such variable spring-damper systems for broader applications involving collisions and conducting comparative experiments with active control methods. Such studies could further highlight the strengths and limitations of passive versus active controllers, providing deeper insights into their respective benefits and trade-offs across various collision scenarios.

## Supplementary Information

Supplementary Information.

## Data Availability

All data supporting the findings of this study are included in this article and its supplementary information file. The raw datasets used and analysed during the current study are available from the corresponding author on reasonable request.
